# Group schema-focused therapy enriched with psychomotor therapy versus treatment as usual for older adults with cluster B and/or C personality disorders: a randomized trial

**DOI:** 10.1186/s12888-018-2004-4

**Published:** 2019-01-15

**Authors:** S. D. M. van Dijk, M. S. Veenstra, R. Bouman, J. Peekel, D. H. Veenstra, P. J. van Dalen, A. D. I. van Asselt, M. L. Boshuisen, S. P. J. van Alphen, R. H. S. van den Brink, R. C. Oude Voshaar

**Affiliations:** 1 Department of Psychiatry, University of Groningen, University Medical Center Groningen, Post office box 30.001, 9700 RB Groningen, The Netherlands; 2 Department of Epidemiology, University of Groningen, University Medical Center Groningen, Groningen, The Netherlands; 3 Lentis, Mental Health Organization, Groningen, The Netherlands; 4 Vrije Universiteit, Brussel, Belgium; 5 Dimence, Mental Health Organization, Deventer, The Netherlands; 6 Van Andel Ouderenpsychiatrie (GGZ Friesland), Leeuwarden, The Netherlands; 7 Mediant Geestelijke Gezondheidszorg, Enschede, The Netherlands

**Keywords:** Schema focused therapy, Psychomotor therapy, Older adults, Group therapy, Personality disorders, Multicenter RCT, Cognitive techniques, Experiential techniques, Design article

## Abstract

**Background:**

Several types of psychotherapy have been proven successful in the treatment of personality disorders in younger age groups, however studies among older patients are lacking. We developed a group schema-focused therapy (SFT) enriched with psychomotor therapy (PMT) for older adults with cluster B and/or C personality disorders. This paper describes the design of a randomized controlled trial (RCT). We will evaluate the (cost-)effectiveness of this therapy protocol in specialized mental health care. We hypothesize that our treatment program is cost-effective and superior to treatment as usual (TAU) in reducing psychological distress and improving quality of life in older adults treated to specialized mental healthcare.

**Methods:**

A multicenter RCT with a one-year follow-up comparing group schema-focused therapy enriched with psychomotor therapy (group SFT + PMT) and TAU for adults aged 60 years and older who suffer from either a cluster B and/or C personality disorder. The primary outcome is general psychological distress measured with the 53-item Brief Symptom Inventory. Secondary outcomes are the Schema Mode Inventory (118-item version) and the Young Schema Questionnaire. Cost-effectiveness analysis will be performed from a societal perspective with the EuroQol five dimensions questionnaire and structured cost-interviews.

**Discussion:**

This study will add to the knowledge of psychotherapy in later life. The study specifically contributes to the evidence on (cost-) effectiveness of group SFT enriched with PMT adapted to the needs of for older adults with cluster b and/or c personality.

**Trial registration:**

Netherlands Trial Register NTR 6621. Registered on 20 August 2017.

## Background

The prevalence rate of personality disorders among community-dwelling older people is estimated at 8% [1] and varies between 33 and 58% among older people referred to specialized mental health care [2–4]. The disease burden due to personality disorders is high for patients (lowered quality of life, high levels of psychological distress and a high suicide risk) as well as for society (increased medical consumption and informal care) [5, 6]. For example, maladaptive personality traits are associated with a 16–30% increase in somatic healthcare consumption among older adults [7, 8]. Adequate detection and treatment is therefore warranted from both a patient and societal perspective. Even in specialized mental health care for older adults, personality disorders often remain undiagnosed and undertreated [9, 10]. Among younger patients several psychological therapies, among which schema focused therapy (SFT) have been developed that effectively reduce the burden of personality disorders [11, 12]. SFT addresses how early maladaptive schemas influence daily life and interpersonal relationships. Schemas pertain to one’s core conceptions of self, others and the world. They are formed in childhood and adolescence. In case of adverse circumstances, maladaptive schemas and associated coping styles will develop to survive (emotionally) which later on, in a healthier environment will lead to interpersonal dysfunctional coping and emotional instability. SFT helps patients to identify their most important maladaptive schemas and to respond in a more adaptive manner when these maladaptive schemas are triggered in daily life.

Review studies in adults (< 60 years) on the treatment for personality disorders concluded that SFT improves quality of life for the borderline personality disorder. They found a recovery from psychological symptoms and that it was less expensive compared to usual care [12]. The aforementioned studies focused on the borderline personality disorder [11] for which SFT was originally developed [13]. However, effectiveness of SFT has also been proven for the avoidant personality disorder [14], mixed group personality disorders [15], and more recently for the chronic mood- and anxiety disorders [16, 17]. Although SFT is generally delivered in an individual format, group SFT is considered to speed up and amplify the effects of individual SFT [18]. Even so-called short-term group SFT [19] is associated with improvements in affective symptoms as well as personality pathology. Studies in younger and middle aged patients which tested the association between the effect of SFT and age did not find a significant impact of age [18, 20]. Extrapolation of these findings to older samples may suggest that SFT also remains effective in later life. Of all psychotherapies focused on personality disorders, SFT is considered most relevant for geriatric practice due to its favorable effects on comorbid, often longstanding affective disorders [21–23]. To date, only three uncontrolled studies of SFT among older adults have been published [9]. These studies point to favorable effects of SFT in later life. First, among 51 depressed inpatients, SFT led to improvement of depressive symptoms, anxiety and five out of the seven maladaptive schemas [24]. Second, among 31 Dutch older outpatients, group SFT improved psychological distress with effect-sizes similar to those reported for younger patients. Moreover, changes in early maladaptive schemas mediated change in psychological distress in this latter study [21]. Third, in a multiple-baseline case series study among 8 older persons suffering from a cluster C personality disorder, psychopathology remained stable during an extended baseline period, while psychopathology significantly diminished during treatment and follow-up [25].

Although these studies among older patients are promising, some caution is needed since no randomized controlled studies evaluating SFT in later life have been conducted yet [9]. Therefore, we decided to perform such a randomized controlled trial and to present its study design here. SFT protocols examined among older patients up to now highly relied on cognitive techniques. Since cognitive techniques may become less effective with increasing age [26], we added several experiential techniques. These techniques are considered powerful methods to change early maladaptive schemas in older adults [21]. Not only did we add experiential techniques to the traditional, verbal SFT approach, we also enriched our treatment protocol with PMT. By offering PMT, patients will experience how their schemas influence their behavior and feelings. The ‘learning by doing’ approach offers opportunities to try out new behaviors [27]. Moreover, discovering the origin of feelings and physical sensations is an important therapeutic mechanism of SFT. PMT contributes to this important ingredient of SFT, by using bodily awareness and physical activities to let patients experience the way they tend to behave [28]. During the preparation phase of this trial, we ran five treatment groups to refine our protocol and test its feasibility. Participants of these pilot groups highly appreciated the psychomotor therapy, the experiential techniques and the adaptation of SFT to a geriatric population, in general.

### Study objectives

The aim of this article is to describe the design of a RCT examining the (cost-)effectiveness of group SFT enriched with PMT compared to treatment as usual for older patients suffering from a cluster B and/or C personality disorder. We hypothesize that our treatment program is cost-effective and superior to treatment as usual (TAU) in reducing psychological distress and improving quality of life in older adults treated to specialized mental healthcare.

## Methods

### Design

We designed a multi-center randomized controlled trial with two parallel treatment groups: 20 sessions of group SFT enriched with PMT delivered over a 6-months period, versus treatment as usual (TAU) for older adults with cluster B and/or C personality disorders in specialized mental health care. Primary outcome is general psychological distress. In addition, cost-effectiveness of the intervention from a societal perspective is evaluated. Outcome measurements will be administered pre- and post-treatment as well as at 6 and 12 months post-treatment. Six mental health care organizations in the Northern Netherlands agreed to participate, one university department of geriatric psychiatry (University Medical Center Groningen) and five affiliated mental health organizations (GGZ Friesland van Andel department, GGZ Drenthe, Lentis, Dimence and Mediant). The study has been approved by the Medical Ethics Committee of the University Medical Center Groningen on May 12th, 2017 (M17.212189) and was registered in the Dutch Trial Register on August 20th, 2017 (NTR 6621).

### Study population

Eligible are patients, 60 years or older with a cluster B and/or C personality disorder according to the DSM-5 who are referred to or are currently treated at an outpatient clinic for geriatric psychiatry of the participating centres in the Netherlands. According to DSM-criteria, the criterion threshold for diagnosing a personality disorder in older patients is too strict [29, 30]. For that reason we will also include older patients falling short one content criterion for a specific cluster B and/or C personality disorder, provided that they meet the general diagnostic criteria for a personality disorder (hereafter ‘subthreshold cluster B/C personality disorder’). Older patients generally endorse fewer specific personality disorder criteria than younger age groups (29% of the criteria contain measurement bias in older age groups), while the latent variable structure for each personality disorder suggests a similar severity level of personality pathology [30]. Specific inclusion criteria are: 1) age of 60 years or above; 2) cluster B or C personality disorder (or falling one content criterion short) as confirmed by the Structured Clinical Interview for DSM-5 for personality disorders (SCID-5-PD) [31] 3) mentally able to adhere to the group SFT treatment schedule and to fill out the schema (mode) questionnaires and 4) giving informed consent after having received oral and written information. Exclusion criteria are: 1) severe current mental illness, including bipolar I disorder, psychosis, or substance abuse disorders needing clinical detoxification; 2) an established neurodegenerative disorder; 3) cognitive impairment defined as a sum score below 23 points on the Montreal Cognitive Assessment (MoCA) battery [32]; 4) having received schema-focused therapy in the previous year or during the current illness episode; and 5) suicide risk interfering with adequate treatment delivery. Physical restraints or physical frailty are no exclusion criterion. Despite the group format, the PMT will be individually adapted so that even the frailest patients can participate.

### Recruitment

**Fig. 1:**
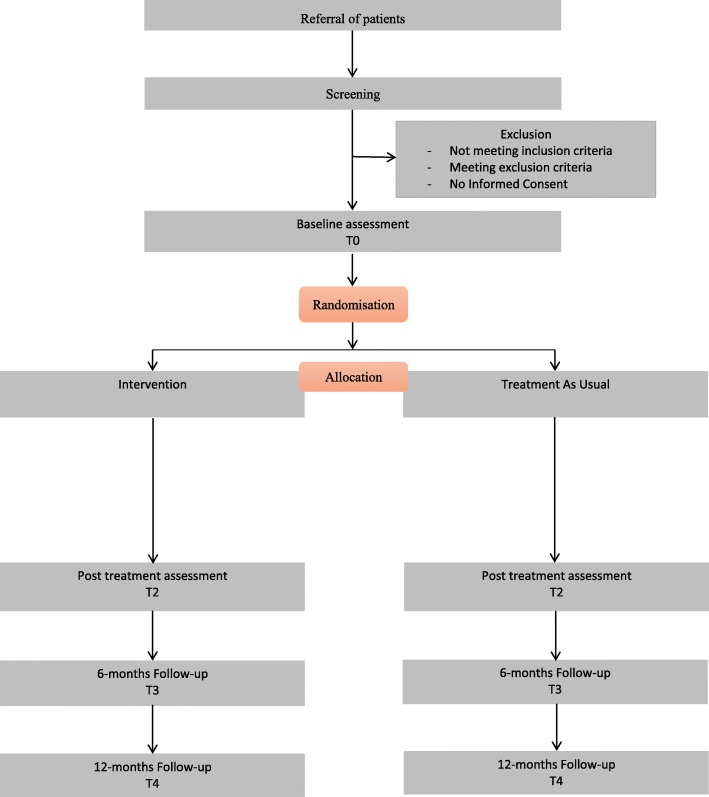
depicts the flowchart of the study

Mental health professionals working at the different sites can inform patients about the study when a cluster B or C personality disorder is suspected. After having received oral and written study information from their mental health professional interested potential eligible patients will get an appointment with the clinical psychologist at their clinic. During this screening session, the in- and exclusion criteria for the study are formally checked by administering amongst others the SCID-5-PD and MoCA and any remaining questions the patient has about the study are answered. For patients not meeting the in- and exclusion criteria the study ends after this screening session. Patients eligible for the study, will be given another 2 weeks to consider participation. Patients willing to participate in the study, will be asked to sign informed consent and are put on a waiting-list. Only the patients who gave written informed consent are included in the study. The data from the screening appointment, will be passed on – anonymously – for inclusion in the study database. When a minimum of 8 and maximum of 16 patients have given informed consent at a specific site, baseline measurements will be conducted, after which participants will be randomized (see flowchart) Fig. 1.

### Sample size calculation

The only RCT comparing group SFT with TAU was conducted among younger adults with borderline personality disorder and found a between group Cohen’s *d* effect size (ES) of 2.0 on the Brief Symptom Inventory (BSI-53) [33]. For our power analysis, we tuned down the expected ES to 0.5 (medium effect) for two reasons. First, the ES of 2.0 was partly due to absence of an effect of TAU [34] while meta-analysis of psychotherapy in geriatric psychiatry shows an average pre-post ES of 0.4 for TAU [17]. Second, an open study on group SFT reported a pre-post Cohen’s *d* ES of 0.8 among older adults with mixed cluster B and C personality disorders [21]. Applying a 2-sided alpha of 0.05 and a power of 80% to detect an ES of 0.5, requires 63 patients per arm. Although compensation for dropout is not necessary according to the CONSORT criteria when performing intention to treat analyses (http://www.consort-statement.org/), we aim to include 140 patients in order to compensate for 10% early dropouts during the waiting-list period. Differences in the scores between the intervention and control group on the various outcome measures (BSI-53, EQ-5D-5 L, YSQ and SMI) will be analyzed using linear mixed-models accounting for missing data and relevant confounders.

### Randomisation, blinding and treatment allocation

A stratified block randomisation will be performed aimed to assign participants evenly (1,1) over the two conditions [35]. Randomisation will be performed per study site and stratified by the presence of a full versus subthreshold cluster B/C personality disorder (PD – 1 criterion). Participants will be randomized in blocks when 8 to 16 consecutive patients have consented to participate at a specific study site. Randomisation will be performed centrally using online randomisation software QuickCalcs of GraphPad (http://www.graphapd.com/quickcalcs/randMenu).) by an independent investigator who will be blind to patient characteristics. Patients will be randomized in even patient numbers per stratum. In case of an uneven number of participants per stratum, the next even number of patients will be randomised and the last allocation will be forwarded to the next block randomisation with an uneven number of patients within that stratum. After randomisation, patients and their responsible geriatric psychiatrist or psychologist will be informed of their allocation. The therapists delivering the intervention will be informed which patients can be invited for group SFT + PMT.

### Group Schema-focused therapy enriched with PMT

Two individual pre-treatment sessions without PMT take place before the group sessions. These are meant to make a personal treatment plan based on administering the Young Schema Questionnaire (YSQ) and Schema Mode Inventory (SMI) (see Mediators section below) and to explain the concept of group SFT enriched with PMT in more detail. The subsequent group interventions comprise 20 sessions over a period of 26 weeks. The first 18 sessions will be provided weekly after which two follow-up sessions are given at week 22 and 26. Each treatment session consists of a 2 h group session SFT and 1 h group session PMT, with a 15-min break in between. Treatment is provided according to a detailed protocol. This protocol is highly structured and based on the Dutch protocols short cognitive schema-focused group therapy [36] and experiential techniques [37, 38]. During the initial group sessions, patients will be further educated about the schema model, specifically in relation to their own three predominant schemas and coping styles (called ‘modes’ in schema focused therapy). In the second treatment phase the patients maladaptive schemas will be triggered and patients will be taught to respond in a more adaptive manner. In this treatment phase the ‘experiential imagery rescripting’ and ‘chair interventions’ will be conducted [37, 38]. All patients receive a personal schema-focussed therapy workbook for older adults. This workbook will be extended every session with a summary of the session information and homework assignments. The patients will write a weekly summary of their therapy process and fill in a ‘severity score form’. On this form patients rate the severity of their 3 predominant schemas and maladaptive modes. Based on clinical experience with older persons, geriatric themes like loss of a role in society, loss of loved-ones, comorbid somatic diseases and sociocultural beliefs in the treatment of elderly, are integrated in the treatment protocol [39, 40]. Furthermore, the protocol has been enriched with PMT to overcome the limitations of verbal therapy. During PMT, the psychomotor therapist sets up individual and group-based physical interventions to facilitate that patients experience their schemas and modes. Continuity between the verbal and psychomotor sessions is guaranteed by participation of the psychologist in the PMT as co-therapist. This way the observations pending PMT can be further analyzed in the verbal sessions.

### Treatment as usual

Treatment as usual (TAU) will be unrestricted as long as no group SFT is provided. Usually this means that a multidisciplinary team will determine the best treatment for each patient. This can be either psychotherapeutic or drug treatment, individual or group treatment, outpatient-, day- or inpatient treatment or any combination deemed necessary. The treatment provided will be registered, to facilitate interpretation of results. From our clinical experience, we expect that TAU will mainly consist of structured and supportive case-management of personality problems or treatment restricted to (comorbid) axis I psychiatric disorders, ideally according to disorder specific guidelines.

### Therapists, training and supervision

All interventions in the experimental group are provided by psychologists with a minimum of 2 years post graduate clinical training in combination with fully licensed and registered psychomotor therapists. During the verbal SFT sessions, a co-therapist will participate. Co-therapists are generally also psychologists but psychotherapeutic oriented nurse practitioners experienced in group treatment are also allowed. One of the psychologists providing verbal SFT will also participate as co-therapist in the PMT sessions. All psychologists and psychomotor therapists receive a two-day training led by the first author, a clinical psychologist who is also a licensed schema-focused therapist. The psychomotor therapists are trained by the third author. This training covers how to deliver a structured SFT program, training of the SFT adaptations to older adults and PMT techniques. The training is supported by video material of verbal SFT and PMT techniques. All therapists receive a manual with the treatment protocol, including a detailed description of all sessions divided in a SFT and a PMT section. Moreover, to improve protocol compliance, the SFT-interventions provided must be ticked off on standardized forms (part of the manual) per session. Session attendance of the individual patients will also be recorded by the therapists. During the study, all therapists will participate in a monthly supervision program provided by the first and third author. Furthermore, all SFT and PMT group sessions in the intervention arm will be audiotaped and saved for evaluation. Of each therapy group two audiotapes will be randomly selected and rated by independent psychologists on protocol compliance. All interventions listed in the treatment manual for the selected session will be evaluated on whether or not the intervention was delivered and the quality of that deliverance, as rated on a scale ranging from 0 (not delivered) to 10 (delivered excellently). A total of at least 10 tapes will be rated by two raters to assess the interrater reliability, as expressed the intra class correlation coefficient (ICC) [41].

### Assessments

Data are collected at the following moments: at the screening, baseline (T1), at the end-of-treatment (T2, i.e. 6 months after baseline for the TAU group), and finally at 6- and 12-month follow-up (T3 and T4). Many outcomes consist of self-report measures. Some are assessed by structured telephone interviews conducted by research assistants who will be blind about the treatment allocation of the interviewed patient. These interviews include assessment of socio-demographic characteristics and psychiatric history (at baseline only) as well as medical consumption and costs in the past 3 months. Personal information about potential and enrolled participants will be stored confidentially. Table 1 shows the questionnaires used at each assessment.

**Table 1:** .shows the questionnaires used at each assessment

	Time of assessment				
	Screening	Baseline	Post treatment assessment	6-month Follow-up	12-month Follow- up
Primary outcomes					
Psychological distress: BSI-53		X	X	X	X
Health-related quality of life: EQ-5D-5 L		X	X	X	X
Medical consumption & costs		X	X	X	X
Secondary outcomes					
Life satisfaction: Cantril’s Ladder		X	X	X	X
Mental wellbeing: WEMWBS		X	X	X	X
Personality functioning: SIPP-SF		X	X	X	X
Interoceptive body awareness: MAIA		X	X	X	X
Psychotropic drug use & treatment received					
Mood variability: Mood-Zoom		X	X	X	X
Characteristics					
Personality disorders: SCID-5-PD Cluster B & C	X				
Mental disorders: MINI-Plus	X				
Cognitive screening: MoCA	X				
Psychiatric treatment history: telephone interview		X			
Socio-demographics: telephone interview		X			
Chronic illnesses: LASA questionnaire		X			
Early life-events: NEMESIS questionnaire		X			
Alcohol use: AUDIT		X			
Current smoking: NEMESIS questions		X			
Physical activity: NEMESIS question		X			
Personality traits: PID-5-SF		X			

### Primary outcome parameter

Psychological distress in the past week, as indicated by the sum score of the Brief Symptom Inventory 53 item version (BSI-53), is the primary clinical outcome parameter. The BSI-53 is a self-report questionnaire and an abbreviated version of the Symptom Checklist-90 (SCL-90) [33, 42]. The BSI is validated for older adults and preferred over the SCL-90 since validation studies in older adults did not indicate any information loss compared to the SCL-90 [43]. We have chosen for psychological distress as the primary outcome parameter since 1) older patients with personality problems are rarely referred to geriatric mental healthcare for personality problems specifically, but nearly always for associated affective symptoms and 2) the study includes patients with a variety of personality disorders. Since the reliability of the Dutch BSI-53 subscales is rated as good and their convergent and divergent validity has been found to be satisfactory [42], subscale scores will be examined as secondary outcome parameters.

### Economic evaluation

The EuroQol-5D-5 L (EQ-5D-5 L), a generic self-report instrument, will be administered to measure health-related quality of life at the time of assessment [44]. It consists of five questions, each related to a specific dimension of health status: mobility, self-care, usual activity, pain/discomfort and anxiety/depression. The utility score of the EQ-5D-5 L will be used to calculate Quality-Adjusted Life-Years (QALYs) [45]. Medical consumption and other cost data will be collected by means of structured telephone interviews, as previously done in a younger patient group [44]. Since a considerable amount of resource use is situated outside (mental) healthcare institutions, data of formal registries such as hospital information systems or insurer’s databases are incomplete [44]. Therefore, patient-reported prospective cost diaries [46] or retrospective cost interviews [47], are the preferred instruments covering all relevant events. We have chosen a 3-month recall interview [48, 49] since a prospective cost diary or a recall interview over a longer period are expected to lead to more missing items.

### Secondary outcome parameters

A number of secondary outcome parameters have been chosen to specify the extent and nature of the effect of group schema focused therapy in later life.

#### Psychotropic drug use

Data on current psychotropic drug use and changes in between assessments will be collected as part of the telephone interviews, described above.

#### Life satisfaction

Life satisfaction will be assessed with Cantril’s ladder, a single self-report question to rate one’s current life situation on a scale (from 0 to 10) [50]. Life satisfaction is a conceptualization of subjective wellbeing which stresses the cognitive evaluation of one’s life situation, in contrast to for example feelings such as happiness. A score of 0 indicates ‘the worst possible life for you’ and 10 ‘the best possible life for you’.

#### Mental wellbeing

Mental wellbeing is assessed with the Warwick-Edinburgh Mental Well-being Scale (WEMWBS) [51]. Mental health and mental illness have been shown to be related but distinct concepts [52]. Reduction or absence of mental illness does not necessarily imply good mental health and wellbeing. The WEMWBS focusses on mental wellbeing and consists of 14 items covering positive affect, satisfying interpersonal relationships and positive functioning. Items have to be rated on a 5-point Likert scales assessing the frequency of the positive feeling over the past 2 weeks. A higher sum score indicates a better mental wellbeing.

#### Personality functioning

Personality functioning is assessed with the Severity Indices of Personality Problems – Short Form (SIPP-SF) [53]. The SIPP-SF assesses five core domains of (mal)adaptive personality functioning defined in the DSM-5 alternative dimensional personality disorders model, namely: Identity Integration, Self-Control, Relational Functioning, Social Concordance and Responsibility. The 60-items of the questionnaire consists of propositions referring to the last three-months, which are answered on four-point Likert scales, ranging from fully agree to fully disagree. Higher scores imply more adaptive functioning. The SIPP-SF has been studied in an older sample and was suggested to be a useful clinical tool to assess effects of therapy on levels of personality functioning in this age group [54].

#### Interoceptive body awareness

Interoceptive body awareness is assessed with the Multidimensional Assessment of Interoceptive Awareness (MAIA) [55]. The MAIA is a 32-item self-report questionnaire, measuring eight dimensions covering both the ability to notice bodily sensations and to regulate their influence on behavior. Treatment responsiveness of the MAIA was recently shown in a study on bodily focused contemplative training [56] which found improvements on five of the eight MAIA scales.

#### Mood variability

Trying to measure emotion dysregulation in real life situations, we will assess mood variability with the self-report Mood Zoom, an experience sampling method for real-time mood assessment on a smartphone [57]. Participants are prompted by the phone to rate their current mood on a screen displaying six different moods which have to be scored on 7-point Likert scales. We will prompt participants three times a day, at random time points, over a one-week period per assessment.

### Patient characteristics (and potential predictors of treatment outcome)

Patient characteristics will be assessed during the screening procedure in order to verify in- and exclusion criteria and the formal baseline assessment, to describe the study sample, and to test and control for baseline differences between the study groups. The screening procedure includes the formal assessment of cluster B or C personality disorders, 2) comorbid mental disorders, and 3) cognitive functioning. The formal baseline assessment includes 4) a psychiatric treatment history, 5) socio-demographics, 6) chronic somatic diseases, 7) early life events, 8) alcohol use, 9) current smoking, 10) physical activity and 11) personality traits.

#### Personality disorders

Personality disorders, according to the traditional categorical DSM-5 model, will be assessed with the Structured Clinical Interview for DSM-5 for personality disorders [31]. To minimize patient burden, the interview will be limited to the general diagnostic criteria for a personality disorder and the cluster B or C personality disorder criteria.

#### Mental disorders

Comorbid psychiatric disorders will be assessed with the aid of a DSM-5 checklist. This checklist summarizes all DSM-5 criteria for the following disorders: Major Depressive Disorder, Persistent Depressive Disorder, Manic and Hypomanic Episodes, Bipolar Disorders type I and II, Generalized Anxiety Disorder, Panic Disorder, Agoraphobia, Social Anxiety Disorder, Posttraumatic Stress Disorder, Obsessive Compulsive Disorder, Somatic Symptom Disorder, and Illness Anxiety Disorder. Based on the screening interview with the patient and all available information in the medical records, the psychologist checks which criteria have been present over the past 6 months and which psychiatric disorders are currently present.

#### Cognitive functioning

Global cognitive functioning will be assessed with the Montreal Cognitive Assessment (MoCA). The MoCA is a screening instrument to be administered by trained interviewers (psychologist in this study) for the detection of neurocognitive disorders and assesses a broad range of cognitive domains. We will exclude patients with a sum score below 23 points. A recent meta-analyses showed that a cutoff score of 23/30 shows overall better diagnostic accuracy and a lower false positive for the presence of neurocognitive disorders than the initially recommended score of 26/30 [32].

#### Psychiatric treatment history

Time of first treatment in mental healthcare will be assessed in the structured telephone interview at baseline.

#### Chronic somatic diseases

The presence of chronic somatic diseases is assessed by self-report questions as validated within the Longitudinal Aging Study Amsterdam (LASA) [58, 59]. The questions inquire about the presence of the following chronic diseases: chronic non-specific lung disease, cardiac diseases, atherosclerotic disease of the abdominal aorta or the arteries of the lower limb, diabetes mellitus, cerebrovascular disease, osteoarthritis, rheumatoid arthritis, malignant neoplasms, high blood pressure, stomach ulcers, bowel disorders, liver disease, epilepsy, allergies, thyroid disease, injuries, serious head trauma, and other chronic diseases. The LASA study shows that the accuracy of patients’ self-reports, as compared to general practitioners’ information regarding to the presence or absence of specific chronic diseases, is generally satisfactory [59].

#### Early life events

Childhood trauma will be assessed using a structured inventory previously used in the Mental Health Survey and Incidence Study (NEMESIS) [60]. In this inventory participants are asked whether they have experienced any kind of neglect or abuse before the age of 16. Covered are: emotional neglect, which includes the lack of parental attention or support and interest in one’s problems and experiences; psychological abuse, which includes verbal abuse, punishment without reason, subordination to siblings and being blackmailed; physical abuse, which includes being kicked, hit with or without an object and any other physical harm; and sexual abuse, which is defined as being sexually touched against one’s will, or being forced to touch someone sexually. After an affirmative answer, a question is asked about the frequency of these events, to be recorded as: never, once, sometimes, regularly, often or very often. A childhood abuse index can be constructed by recoding the frequency scores in (0) never, (1) once, sometimes and (2) regularly, often or very often. The scores of each of the four domains of abuse will be summed, resulting in a childhood abuse index which ranges from 0 to 8. Early life-events will be additionally measured and include divorce of parents, placement in children’s home, juvenile prison or foster care, and walking away from home.

#### Lifestyle characteristics

Alcohol use will be assessed with the Alcohol Use Disorder Identification Test (AUDIT) [61], a screening questionnaire for alcohol-related problems in the past year. Current smoking and physical activity will be assessed by two single questions from NEMESIS on current smoking and number of hours per week spent in physical exercise or sport lately, not counting sedentary pursuits such as chess or fishing [60].

#### Personality traits

Pathological personality traits are a second dimension of the DSM-5 alternative dimensional model for personality disorders and can be measured with the 220-item Personality Inventory for DSM-5 (PID-5) [62]. The PID-5 covers 25 pathological personality trait facets, which can be combined to obtain scores for five higher-order domains distinguished. These domains are related to the traditional big five personality traits and to concepts from schema-focused therapy [63]. Most items of the PID-5 are age neutral and thus can be used in older adults [64]. Recently, the PID-5-Short Form (PID-5-SF) has been validated, yielding nearly identical reliability and validity as the long form in scoring the DSM-5 domains and facets [63]. This version will be applied in the present study to minimize patient burden. All items are rated on four-point Likert scales from 0 (very false or often false) to 3 (very true or often true). The combination of pathological personality traits (PID-5-SF) and impaired personality functioning (SIPP-SF) results in the alternative DSM-5 model personality disorder diagnoses.

### Mediators

We will examine whether any improvement on the primary and secondary outcome parameters in patients receiving the experimental schema therapy can be attributed to (i.e. is mediated by) improvements in the therapy specific targets. Mediators are early maladaptive schemes and schema modes, which will be assessed,in the experimental group only, pre-treatment (i.e. in the two initial, individual sessions of treatment) and 3, 6 and 12 months post-baseline, and schemas and modes severity scores, which will be assessed during treatment on a weekly basis.

#### Early maladaptive schemas

Early maladaptive schemas will be assessed with the self-report Young Schema Questionnaire second version (YSQ-2) [65–68]. The YSQ-2 is the most commonly used measure of early maladaptive schemas [65]. The YSQ-2 consists of 205 items, to be rated along a 6-point Likert scale, which measures the severity of 16 maladaptive schemas. The Dutch YSQ-2 has good reliability and convergent and discriminant validity [69].

#### Schema modes

Schema modes will be assessed with the Schema Mode Inventory (SMI), a 118-item, self-report questionnaire measuring 16 modes [65]. These modes can be divided into 4 types: healthy modes, parent modes, child modes and coping modes. All items are rated on a 6-point Likert scale. The Dutch SMI has an excellent test-retest reliability and the convergent and divergent validity of the subscales are satisfactory [70].

#### Schema and modes severity scores

Patients will rate the severity of their three most dominant schemas and modes on a scale from 0 (no burden at all) to 10 (a lot of burden) on a weekly basis.

### Analyses

Analyses will be conducted according to the intention-to-treat principle. Differences between the intervention and control group on the various outcome measures (BSI-53, EQ-5D-5 L, secondary outcome parameters) will be analyzed using linear mixed-models, more specifically random coefficient analysis, which accounts for missing observations [71]. In these analyses, study site and patient will be included as random effects (with observations nested in patients and patients nested in study sites) and study arm and full versus subthreshold personality disorder as fixed effects. Interactions will be tested 1) between time of observation and study arm and 2) between this interaction and full versus subthreshold personality disorder, to check whether the intervention effect is different for patients with a full or subthreshold personality disorder. All analyses will be controlled for the baseline population characteristics described above.

### Economic evaluation and budget impact analysis

The cost-effectiveness analysis will be performed alongside the clinical trial to assess the cost-effectiveness of group SFT + PMT versus TAU. The cost-effectiveness analysis will result in two separate incremental cost-effectiveness ratios (ICERs) for group SFT + PMT compared to TAU, i.e. incremental costs per additional point improvement on the BSI-53, and incremental costs per Quality Adjusted Life Year (QALY) gained. The analysis will be performed taking a societal perspective and with a time horizon of 18 months. Costs and effects will be discounted according to Dutch guidelines [45]. Univariate sensitivity analyses will investigate the impact of individual assumptions and parameters (e.g. costs of SFT + PMT and other elements). To quantify the uncertainty around the ICERs, bootstrap resampling will be performed. Results will be presented as tornado diagrams and cost-effectiveness acceptability curves, the latter representing the probability that group SFT + PMT is cost-effective given a certain value of ‘willingness to pay’ for a QALY (or one point improvement on the BSI). Included costs will be those of group SFT enriched with PMT, other forms of psychotherapy, hospital admissions, medication, outpatient visits, General practitioner visits, home care, and lost productivity from both paid and unpaid work. Unit prices will be determined according to Dutch guidelines [45].

A budget impact analysis will be performed to inform decision makers on the financial consequences of the adoption and diffusion of group STF + PMT in the Dutch health care system. The analysis will be performed according to the most recent principles of the ISPOR task force [72] and Dutch guidelines [45]. The trial results will be extrapolated to a time horizon of 5 years, and for the entire Dutch population concerned. Aging of the population will be taken into account. Sensitivity analysis will explore several scenarios, regarding speed of uptake of the therapy in clinical practice and patients’ willingness to participate.

## Discussion

This paper presents the design of the first randomized controlled trial evaluating the effectiveness of group SFT enriched with PMT, for older adults with personality disorders. To date, only uncontrolled studies on the treatment of personality disorders have been conducted in this age-group [21, 25]. This RCT is especially relevant in the light of aforementioned underdetection and undertreatment of personality disorders in later life [10]. Nonetheless, we faced many choices when designing the protocol, since group SFT has rapidly evolved over the past decades and its applications have even broadened beyond personality disorders alone. Below the most important choices will be discussed.

### Group versus individual format

Evaluation of group therapy within a randomized controlled design may result in some difficulties. First, sufficient patients have to provide informed consent before the therapy can start. This may lead to early dropouts when recruitment is slow at some study sites and waiting period becomes long. Secondly, some patients are reluctant towards group therapy. This may be especially so for older people, who easily feel they have to ‘hang themselves out to dry’ [73]. Nonetheless, we have taken the risk of non-participation as delivering SFT in a group format has many advantages. First, a group process may speed up the therapeutic process [74]. Secondly, group therapy may counterbalance loneliness, a frequently encountered problem in later life [75] especially among personality disordered persons. Thirdly, a group format is more efficient from an economic perspective [76] as well as in light of the scarcity of gerontopsychologists [77].

### Choice of control group

The choice of an adequate control group is critical when designing a randomized controlled trial. Since personality disorders usually remain largely undetected in geriatric psychiatry and treatment is generally focused on the manifest affective symptoms, we considered comparison to usual care as most appropriate. Even if the mere provision of attention would explain a positive effect of our therapy, this may imply that treatment intensity in geriatric psychiatry in general is too low. Alternative options were disqualified for the following reasons. Firstly, an active control group to control for attention is generally desired in psychotherapeutic research [78]. Ideally, two effective therapies would be compared searching for the most effective treatment modality. However, for none of the available therapies for personality disorders, effectiveness is proven in geriatric psychiatry. When no differences are found then, this could easily lead to the wrong conclusion that both therapies are probably non-effective, and to withholding of effective treatment from older patients with personality disorders. Secondly, a waiting-list control group was dismissed for two reasons. First, the placebo-effect or spontaneous recovery may even be reduced by a waiting list [79]. Second, personality pathology is associated with a high disease burden and when referred to specialized mental health care, a waiting list of 18 months was not deemed acceptable. Finally, from an economic perspective, it is always more informative to assess cost-effectiveness of an intervention as compared to care as usual, since this best approaches the real-world situation if the intervention were to be implemented in routine clinical practice.

### Enrichment with PMT.

Arguments for the addition of PMT have already been given in both the introduction and methods of this article. Nonetheless, this step is probably most debatable as even among younger patients no examples of this combination have been reported. However, pure cognitive interventions are generally less effective in later life [80] and older persons generally have more difficulties in expressing their emotions and feelings verbally than younger persons [81]. In our protocol we added PMT and experiential techniques and provided it to 5 pilot groups who reported a potential surplus value for the experiential techniques and PMT. The continuity of one of the group therapists who also attends the PMT sessions was highly appreciated.

### Primary outcome parameter

Although group SFT focuses on personality change, we did not choose personality (dys)functioning as primary outcome parameter for several reasons. First, we did not restrict our study population to one personality disorder specifically. Many studies on SFT are restricted to borderline personality disorder. In those studies, it is rational to consider the severity of borderline personality pathology as the primary outcome parameter. In studies with mixed personality disorders, however, this becomes less suitable. Moreover, most personality pathology instruments are multidimensional and therefore difficult to consider as primary outcome parameter. And taking recovery, defined as not meeting the DSM-criteria for a personality disorder anymore, as the overarching outcome measure, would mean that a semi-structured diagnostic interview should be repeated at each follow-up assessment, which is time-consuming and could increase patient dropout. Second, in geriatric psychiatry most patients with a personality disorder are referred to specialized mental health care for disabling affective symptoms [30]. Specific treatment for personality disorders is often not available and rarely offered in old age psychiatric services. So the majority of older patients with personality disorders receive pharmacotherapy with or without nurse-led supportive care for an affective disorder, although psychotherapy is the preferred treatment for the underlying personality disorder according to clinical guidelines [45]. This is a situation which calls for a change, as meta-analyses among younger age groups have shown that comorbid personality disorders worsen the prognosis of affective disorders and predict relapse [82]. Moreover, previous studies on SFT in both younger and older patients have reported large effect-sizes for improvement of general distress [83]. Earlier research focused mainly on the cognitive variant of (group) SFT for treatment of personality disorders in younger and middle-aged adults (< 60 years). This cognitive variant may be somewhat less effective for older adults (> 60 years). This study will provide an answer to the question whether group SFT enriched with PMT adapted to the needs of for older adults with cluster b and/or c personality is effective and cost-effective. Acknowledging the numerous methodological choices that have to be made, we hope to motivate other researchers to conduct also RCT’s on the (cost-)effectiveness of the different psychotherapies in older adults with personality disorders.

## Abbreviations

AUDIT: Alcohol Use Disorder Identification Test
BIA: Budget Impact Analysis
BSI: Brief Symptom Inventory
CEA: Cost-Effectiveness Analysis
CERs: Incremental cost-effectiveness ratios
DSM: Diagnostic and Statistical Manual of Mental Disorders
EMS: Early Maladaptive Schemes
EQ-5D-5 L: EuroQoL assessment of health related quality of life
ES: Effect Size
GP: General Practitone
LASA: Logitudinal Aging Study Amsterdam
MAIA: Multidimensional Assessment of Interoceptive Awareness
METC: Medical research ethics committee
MoCA: Montreal Cognitive Assessment
NEMESIS: Netherlands Mental Health Survey and Incidence Study
PD: Personality Disorders
PID-5-SF: Personality Inventory for DSM5 - Short Form
PMT: Psycho-Motor Therapy
QALYs: Quality-Adjusted Life-Years
RCT: Randomized Clinical Trial
RGOc: Rob Giel Research center of Mental Health Services
ROMGPS: Routine Outcome Monitoring for Geriatric Psychiatry & Science
SCID-5: Structured Clinical Interview for DSM-5 personality disorders
SCL: Symptom Check List
SFT: Schema Focussed Therapy
SIPP-SF: Severity Indices of Personality Functioning – Short Form
SMI: Schema Mode Inventory
TAU: Treatment As Usual
Wbp: Personal Data Protection Act (in Dutch: Wet Bescherming Persoonsgevens)
WEMWBS: Warwick-Edinburgh Mental Well-being Scale
